# Dobutamine Alters the Pharmacokinetic and Pharmacodynamic Behavior of Esmolol

**DOI:** 10.7759/cureus.12217

**Published:** 2020-12-22

**Authors:** Günther Krumpl, Ivan Ulč, Michaela Trebs, Juri Hodisch, Pavla Kadlecová, Bernhard Husch

**Affiliations:** 1 Pharmacology, MRN Medical Research Network, Vienna, AUT; 2 Pharmacology, Center for Pharmacology and Analysis (CEPHA) s.r.o, Plzeň, CZE; 3 Operational Business Development, AOP Orphan Pharmaceuticals AG, Vienna, AUT; 4 Drug Safety, AOP Orphan Pharmaceuticals AG, VIenna, AUT; 5 Biostatistics, Advanced Drug Development Services (ADDS) s.r.o, Brno, CZE; 6 Research and Development, AOP Orphan Pharmaceuticals AG, Vienna, AUT

**Keywords:** cardioselective β-blocker, esmolol, dobutamine, pharmacokinetics, pharmacodynamics

## Abstract

Background and objective

This study involved an investigation into the pharmacokinetic and pharmacodynamic behavior of esmolol in the presence of dobutamine in healthy subjects of European ancestry.

Methods

We conducted a single-center, prospective randomized study of 16 healthy subjects with each receiving an infusion of dobutamine sufficient to increase heart rate (HR) by 30 beats per minute (bpm) followed by a 60-minute infusion of 50 µg/kg/min esmolol. Pharmacokinetics, HR, and blood pressure were evaluated for 180 minutes.

Results

In the presence of dobutamine, esmolol elimination was substantially faster than without dobutamine, Esmolol infusion reduced dobutamine-induced elevation of HR reversibly whereas the dobutamine-induced systolic blood pressure (SBP) reduction did not recover after the termination of the esmolol infusion. No serious adverse events (AEs) were observed.

Conclusions

The accelerated elimination of esmolol was likely due to higher cleavage through tissue esterases induced by dobutamine-induced increased tissue passage cycles per time unit. The HR effect was characteristic of a beta-blocker, whereas the blood pressure effect was likely due to a mechanism other than direct beta-blockade. HR remained elevated after the infusion of esmolol and dobutamine, most likely due to persistent blood pressure reduction.

## Introduction

β-Adrenoceptor antagonists are effective regulators of heart rate (HR) in cases of sinus and supraventricular and ventricular tachycardia. Esmolol is an ultrashort-acting β_1_‑adrenoceptor antagonist for parenteral use with a high cardioselectivity (K_i_ β_1_/K_i_ β_2_ = 34) [1]. It inhibits the positive chronotropic effects of the catecholamines adrenaline and noradrenaline on the heart, where β_1_-receptors are predominantly located. In addition, esmolol also acts as an inhibitor of sodium (Na) and calcium (Ca) channels and blocks potassium (K) channels, which is thought to be the reason for its negative inotropic effect [2-5]. Its antiarrhythmic and anti-ischemic effects are thought to be due to the reduction of the sympathetic drive, which results in HR reduction, decrease in spontaneous firing of ectopic pacemakers, as well as slowing down of the electrical conduction of the atrioventricular (AV) node, and the increase in its refractory period. Also, exposure to esmolol amplifies the re‑expression of β-receptors [6], which contributes to the drug tolerance effect seen during long-term esmolol infusion [7,8].

Esmolol is a well-studied drug exhibiting typical β-blocker hemodynamic and electrophysiologic effects [9,10]. It is much more selective than other available intravenous (IV) β-blockers such as metoprolol [9]. With a reported *in vivo* half-life of nine minutes [11], esmolol is used for the rapid control of the ventricular rate in emergencies and in patients with perioperative tachycardic arrhythmias, tachycardia, and/or hypertension. In addition, it has been studied for treatment or prophylaxis of relative or absolute tachycardias in the perioperative phase with positive [12] or less positive [7,13,14] results. Further studies have been undertaken in unstable angina and acute myocardial infarction [15].

According to its summary of product characteristics (SPC) (see prescribing information for Brevibloc: http://brevibloc.com), the IV dosage schedule of esmolol is not particularly convenient and makes appropriate dosage administration cumbersome. Especially, different dose recommendations for different settings and the complicated staircase titration of 500 and 50 µg/kg/min are prone to confuse the users.

Across the dosage range used in the clinical setting, steady-state blood concentrations of esmolol increase linearly, and elimination kinetics is reported to be not dose-dependent [11,16]. Esmolol is metabolized by esterases into an acid metabolite and methanol [1,17]. Since the half-life of esmolol is much longer in plasma than in whole blood, it has been assumed that these esterases are located in red blood cells [18]. However, *in vitro* studies have shown that the half-life of esmolol in human whole blood varies between 19.6 and 27.2 minutes at 37 °C [19]. Based on the fact that these *in vitro* values are still higher than the *in vivo* half‑life of nine minutes observed in humans, we and others [20] assume that esmolol is to a higher extent metabolized by extravascular enzymes, as implied by its higher volume of distribution of around 3 l/kg.

Dobutamine is a β-adrenoceptor agonist (predominantly β_1_) with an inotropic effect. This sympathomimetic drug is commonly used as a cardiac stimulant in the treatment of heart failure and cardiogenic shock. It is also used in cardiac stress testing to help identify coronary artery disease, as an alternative to physical exercise in patients who cannot perform routine workouts in a satisfactory manner [21]. Because of its short half-life, dobutamine is administered as a continuous IV infusion.

In our prospective cross-over study in healthy Caucasian volunteers, we investigated the pharmacokinetic and pharmacodynamic characteristics of a permanent infusion of esmolol or landiolol during dobutamine challenge. We describe for the first time the pharmacodynamic and pharmacokinetic behavior of esmolol at the lowest recommended standard dose (50 µg/kg/min) during dobutamine challenge; the results obtained with landiolol are presented separately [22].

## Materials and methods

Materials

Esmolol hydrochloride infusion solution (Brevibloc® 2,500 mg esmolol/250 ml) was obtained from Baxter Vertriebs GmbH (Vienna, Austria). Dobutamine (Admeda 250 solution for infusion, 50 ml; concentration: 5 mg/ml; Admeda Arzneimittel GmbH, Nienwohld, Germany) was diluted to 1 mg/ml with sterile isotonic saline prior to use.

Study approval

We performed a prospective single-center, prospective, double-blinded, randomized study with two cross-over periods in compliance with the respective study protocol, the Declaration of Helsinki, Good Clinical Practice (GCP) and Good Laboratory Practice (GLP) guidelines, as well as other applicable international and national regulatory requirements and local laws after obtaining approval of the study protocol by the Ethics Committee. The study was registered at EudraCT (2010-023311-34). All study participants gave written informed consent before the conduct of the study-related procedures.

Study population

Sixteen healthy volunteers [as determined by medical history, physical examination, electrocardiogram (ECG), 2D-echocardiography, hematology, coagulation, clinical chemistry, serology, urinalysis, and testing for drug abuse at the screening] of European ancestry were recruited for the study. Inclusion criteria were as follows: age of 18-45 years (inclusive), a body-mass index (BMI) of 18.5-30.0 kg/m^2^ (inclusive), non-smoking, ex-smoking (completely stopped smoking for at least three months) or mild smoking (nine cigarettes or less per day). Exclusion criteria were, among others, a history or presence of clinically relevant cardiovascular, renal, hepatic, ophthalmic, pulmonary, neurological, metabolic, hematological, gastrointestinal, endocrine, immunological, psychiatric, or skin diseases, hypersensitivity to landiolol, esmolol, dobutamine, or related drugs, as well as pregnancy and/or breast-feeding. Individuals with inappropriate vascular anatomy (small, badly visible, or invisible veins) were also excluded.

Study conduct

Our prospective, double-blinded, randomized, two-period, two-treatment crossover study aimed at comparing the short-term pharmacokinetics, pharmacodynamics, and tolerability of landiolol with that of esmolol in the continuous presence of the adrenergic stimulant dobutamine in healthy volunteers; it was conducted between March and December 2011 at the Center for Pharmacology and Analysis (CEPHA s.r.o.), Plzeň, Czech Republic. Participants were assigned to one of two treatment sequences (landiolol/esmolol or esmolol/landiolol) using a predefined 1:1 randomization scheme. The duration of the treatment periods was two days each, with confinement from at least 11 hours before and until at least eight hours after the end of study drug administration, and a washout phase of two days minimum between periods. The end-of-study examination was performed within 72 hours after the end of infusion.

Three indwelling catheters were placed into the cubital veins of each subject in each period. One catheter was used for dobutamine infusion and the other at the same arm for study drug infusion. Blood sampling was performed from the third catheter on the arm contralateral to the study drug administration site. Drug administration and sampling sites were switched between periods. Study subjects were required to maintain bed rest and supine position for safety reasons from the start of dobutamine infusion until two hours after the end of the study drug administration.

Dobutamine infusion was initiated at 10 μg/kg/min for 10 minutes and incremented by 5 μg/kg/min every 10 minutes until the targeted HR increase of at least 30 beats per minute (bpm) above baseline or a maximum dose of 30 μg/kg/min was reached. Once the target HR was established, dobutamine administration was continued at an unchanged rate for another 20 minutes before esmolol (dose: 50 μg/kg/min) or landiolol (10 µg/kg/min) was administered for 60 minutes. Dobutamine infusion was continued at an unchanged rate during and after β-blocker infusion until the HR had returned to a maximum value or to the value before β-blocker infusion or until 60 minutes after the end of the β-blocker infusion, whichever occurred first. ECG was monitored continuously from 10 minutes before initiation of dobutamine infusion until at least 120 minutes after the end of β-blocker infusion. ECG parameters (PQ, QRS, QT) were checked throughout at regular intervals.

Measurements

Blood samples were collected at time 0 (before the start of β-blocker infusion), and at 2, 4, 6, 8, 12, 16, 20, 28, 36, 44, 60 (end of infusion), 62, 64, 66, 68, 72, 76, 80, 88, 96, 104, 120 minutes, and 3, 5, 7, and 9 hours after the start of the infusion. HR values (derived from the signal of the bedside ECG monitor) were recorded at the time points for blood sampling and at 15-minute intervals thereafter until 180 minutes after the start of β-blocker infusion. Blood pressure [systolic blood pressure (SBP) and diastolic blood pressure (DBP), measured by the cuff method on the arm used for blood sampling] values were recorded at the start of dobutamine infusion, at 2, 4, 6, and 10 minutes of each dobutamine dose, at 2, 4, 6, 10, 15 and 20 minutes of the maintenance dobutamine dose, and at the start (0) of and at 4, 8, 16, 28, 44, and 60 minutes during study drug infusion. Further recordings were taken 64, 68, 76, 88, 104, 120, 135, 150, 165, and 180 minutes after the start of β-blocker administration.

Assessment of tolerability and safety

Local tolerability was assessed at the start and at the end of β-blocker infusion, and two hours after the end of infusion as described earlier [23]. For the assessment of safety and tolerability, clinically relevant abnormalities in physical examination, vital signs, ECG, laboratory parameters, as well as local tolerability results and adverse events (AEs) were taken into account.

Analytical procedure

Concentrations of esmolol and its metabolite EM in supernatants of ethanol-precipitated whole blood were quantitated using a validated high-performance liquid chromatography-tandem mass spectrometry (HPLC-MS/MS) method as described earlier [8]. The lower and upper limits of quantitation with this method were 1 ng/ml and 550 ng/ml, respectively for esmolol, and 55 ng/ml and 27.8 µg/ml, respectively for EM. Intermediate precision (RSD) across the range of quantitation was <8% and <9% for esmolol and EM, respectively. Accuracy across the range of quantitation was between 93.9% and 107% for esmolol and 94.2% and 105.9% for EM. Stability of ethanol-precipitated whole blood samples has been shown for 23 hours at bench-top temperature, for 168 hours under auto-sampler conditions (5 °C), and for 38 weeks when stored at ≤70 °C.

Pharmacokinetic analysis

Standard pharmacokinetic parameters for esmolol and its metabolite were estimated using non-compartmental as well as one- and two-compartmental models with the validated software package Phoenix^TM^ WinNonlin® version 6.1 (Pharsight Corp., St. Louis, MO).

Pharmacodynamic analysis

Time courses of the pharmacodynamic variables were evaluated in terms of absolute values and changes from baseline on an individual basis and by using descriptive statistics for each data point.

Statistical analysis

Statistical analyses were performed using the software package SAS version 9.2 (SAS Institute Inc., Cary, NC).

## Results

Study population

The baseline demographic characteristics of the study populations are summarized in Table 1. No substantial differences were notable between the groups.

**Table 1:** Baseline demographic characteristics of study populations SD: standard deviation; BMI: body mass index

Demographic variable	Inclusion criterion	Esmolol + dobutamine (n = 16)	Esmolol long-term (n = 14) [8]
Ethnicity	Caucasian White	Caucasian White	Caucasian White
Gender	Both		
Female, n (%)		8 (50.0)	7 (50.0)
Male, n (%)		8 (50.0)	7 (50.0)
Age (years; mean ± SD)	18–45	33.1 ± 8.2	34.4 ± 8.9
Weight (kg; mean ± SD)	50–90	75.9 ± 8.2	73.6 ± 8.7
Height (cm; mean ± SD)	-	176.9 ± 7.0	173.1 ± 8.5
BMI (kg/m²; mean ± SD)	18.5–30	24.2 ± 1.8	24.6 ± 2.4
Smoking habit	≤9 cigarettes or ≤2 cigars/pipes per day		
Non-smoker, n (%)		12 (75.0)	11 (78.6)
Mild smoker, n (%)		4 (25.0)	3 (21.4)

Maintenance dobutamine dose

The mean dobutamine maintenance dose of 16.6 µg/kg/min led to a median HR increase by 43 bpm.

Pharmacokinetics

**Figure 1:**
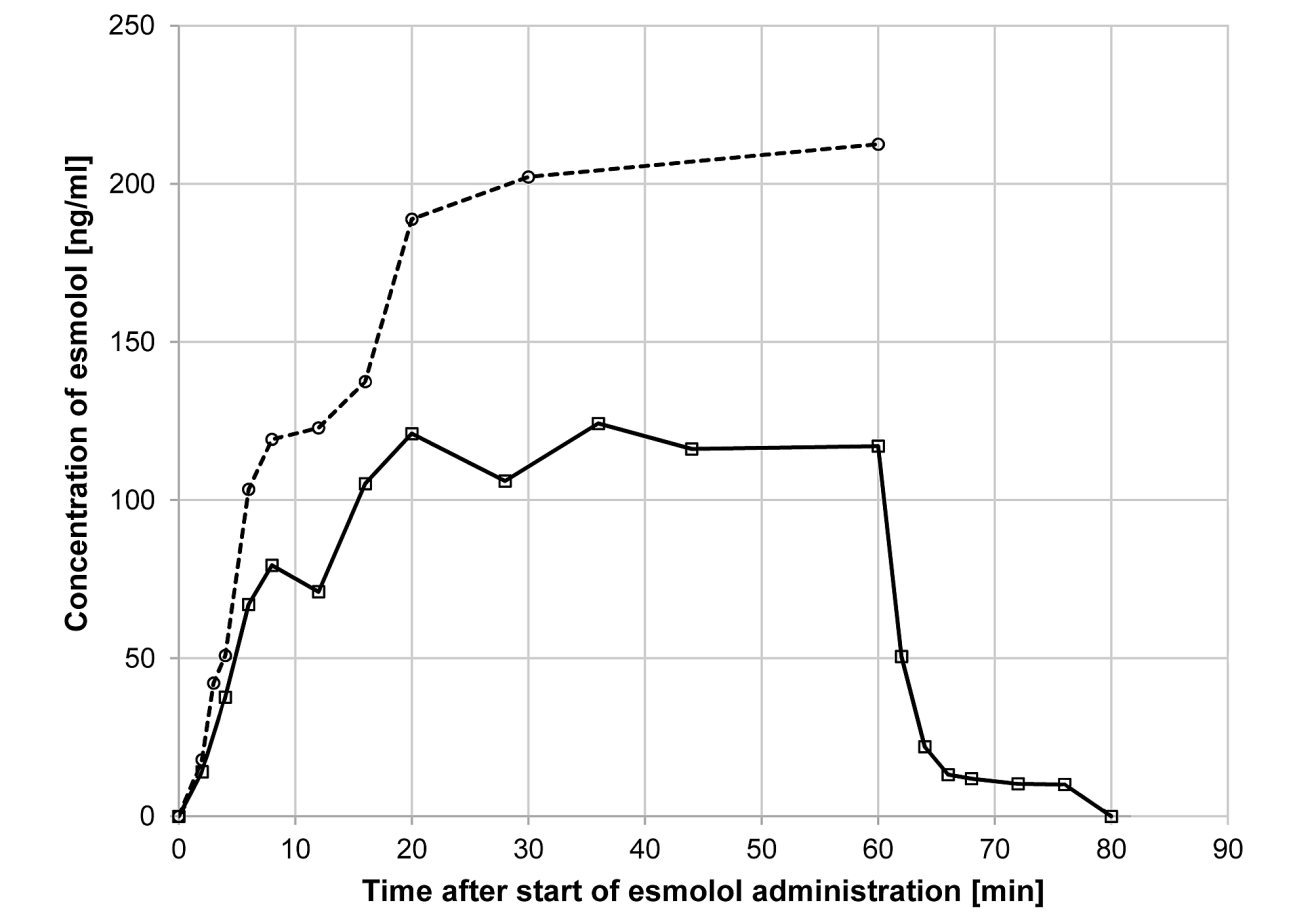
Time course of esmolol concentrations in whole blood in the presence and absence of dobutamine Open squares and solid line: infusion of esmolol (50 µg/kg/min) on top of continuous dobutamine infusion; open circles and broken line: infusion of esmolol (50 µg/kg/min) only [8]. Please note that the elimination phase is lacking for the esmolol-only experiment, because infusion was continued at higher doses for an additional 23 hours

Figure 1 shows the time courses of the blood concentrations of esmolol in the dobutamine group (n = 16) and 12 subjects in another study that received the same esmolol dose (50 µg/kg/min) without dobutamine for two hours followed by infusion with higher dosages of esmolol (100 and 200 µg/kg/min) for up to 24 hours [8]. For this reason, the elimination phase under comparable conditions is not available.

The curve obtained with esmolol infusion in the continuous presence of dobutamine differs substantially from the curve obtained during infusion of esmolol only. The latter shows a later steady-state plateau, whereas, under dobutamine, esmolol achieves an earlier steady-state, a lower c_max,_ and a lower area under the curve (AUC). Esmolol concentrations decreased rapidly after the termination of infusion.

Table 2 summarizes the pharmacokinetic parameters obtained for esmolol when administered in the presence or absence of a continuous dobutamine challenge.

**Table 2:** Pharmacokinetic parameters (noncompartmental analysis) of esmolol in the presence and absence of dobutamine in healthy Caucasians *Data for the first hour of continuous infusion; elimination phase after 20 h infusion at 200 µg/kg/min; ^†^n = 15; ^§^estimated as the arithmetic mean of concentration values obtained after 5 t_1/2_ c_max_: maximum blood concentration; t_max_: time of maximum blood concentration; c_ss_: steady-state concentration; AUC_0-1 h_: area under the blood concentration-time curve from 0 h until 1 h; AUC_0-t_: area under the blood concentration-time curve from 0 h to the last measurable concentration value; AUC_0-∞_: area under the blood concentration-time curve from 0 h to infinity; λ_z_: terminal elimination rate constant; t_1/2_: elimination half-life during the terminal phase; t_lag_: time until first measurable concentration; CL: total clearance; V_D_: volume of distribution; 95% CI: 95% confidence interval; NA: not applicable; SD: standard deviation; geo mean: geometric mean

Parameter	Statistic	Esmolol (50 µg/kg/min) for 60 minutes in the presence of dobutamine (n = 16)	Esmolol (50 µg/kg/min) for 60 minutes in the absence of dobutamine (n = 14)* [8]	Esmolol (200 µg/kg/min) for 20 hours in the absence of dobutamine (n = 14)* [8]	
c_max _(ng/ml)	Geo mean (SD)	151 (1.49)	250 (1.41)	1,290 (1.32)	
	95% CI	122–186	205–305	1,100–1,520	
t_max_ (min)	Median	20	20		
	Range	6.0–44	6.0–60		
t_lag_ (min)	Median	4.0	3.0	NA	
	Range	2.0–12.0	2.0–6.0	NA	
c_ss _(ng/ml)^§^	Geo mean (SD)	115 (1.44)	203 (1.31)	764 (1.48)	
	95% CI	94.9–140	174–237	609–958	
AUC_0-1 h_ (ng.min/ml)	Geo mean (SD)	6,260 (1.43)	10,700 (1.40)	51,500 (1.42)	
	95% CI	5,180–7,560	8,830–13,000	42,100–63,100	
AUC_0-t_ (ng.min/ml)	Geo mean (SD)	6,540 (1.43)			
	95% CI	5,400–7,920			
AUC_0-∞_ (ng.min/ml)	Geo mean (SD)	6,660 (1.44)^†^			
	95% CI	5,400–8,160^†^			
t_1/2 _(min)	Geo mean (SD)	2.85 (1.92)^†^		7.13 (1.47)	
	95% CI	1.98–4.09^†^		5.71–8.92	
λ_z_ (1/min)	Geo mean (SD)	0.244 (1.92)^†^		0.097 (1.47)	
	95% CI	0.17–0.35^†^		0.078–0.121	
CL (ml/kg.min)	Geo mean (SD)	432 (1.47)^†^	243 (1.31)	262 (1.48)	
	95% CI	349–534^†^	208–284	209–328	
V_D_ (ml/kg)	Geo mean (SD)	1,770 (1.76)^†^	2,500 (1.57)	2,690 (1.67)	
	95% CI	1,300–2,430^†^	1,930–3,260	1,810–3,220	

In the presence of dobutamine, the following parameters were obtained for esmolol: steady-state was reached at about 20 minutes, with a c_ss_ value of 115 ng/ml. The maximum blood concentration of esmolol (151 ng/ml) was observed after 20 minutes. After the discontinuation of administration, esmolol disappeared very fast from the bloodstream with a t_1/2_ of 2.9 minutes. The volume of distribution was 1,770 ml/kg, and the total clearance was 432 ml/kg.min. The corresponding values in the absence of dobutamine were c_ss_ = 203 ng/ml, c_max_ = 250 ng/ml, T_max_ = 20 minutes, CL = 243 ml/kg.min and V_D_ = 2,500 ml/kg, respectively. The elimination half-life (obtained after infusion for 24 hours) was 7.1 minutes. In the presence of dobutamine, c_ss_ decreased by 53%, c_max_ by 40%, t_1/2_ by 60%, and AUC by 42% compared to the values obtained in the absence of dobutamine. The distribution volume decreased by 29% while total clearance was increased by 78%. It is worth noting that the ratios of distribution volume and total clearance were not substantially different when compared to the values obtained with the administration of esmolol at 50 or 200 µg/kg/min.

Pharmacodynamics

**Figure 2:**
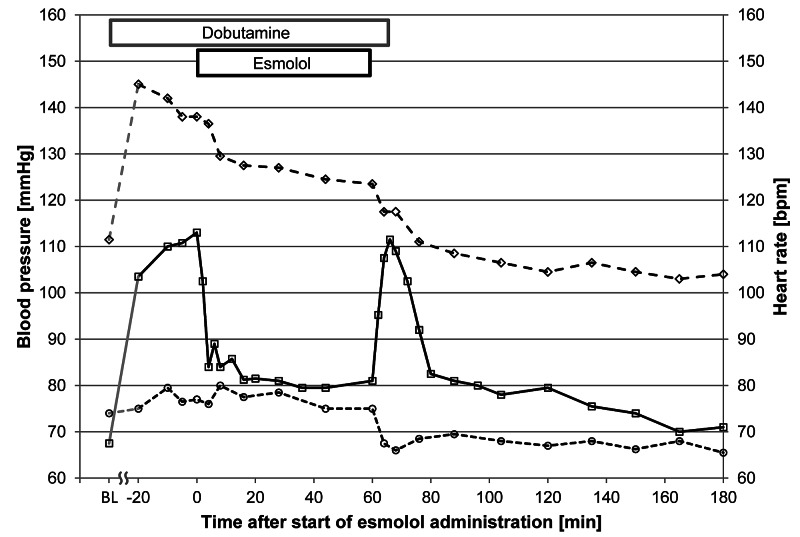
Time course of heart rate, and systolic and diastolic blood pressure Open squares and solid line: heart rate; open diamonds and broken line: systolic blood pressure; open circles and dotted line: diastolic blood pressure BL: baseline

Figure 2 shows the time courses of HR, and SBP, and DBP (all values median). After the initial increase from 67.5 to 113 bpm due to the administration of dobutamine, HR showed a precipitous decline (from 113 to 85 bpm within four minutes) in response to esmolol. The maximum reduction in HR was observed after 18.6 ± 4.9 minutes. Between 16 minutes after the initiation of administration and the end of the administration, HR values were stable around 80 bpm. After the discontinuation of esmolol infusion, a pronounced rebound of HR occurred. The maximum median value of 111.5 bpm was recorded six minutes after the discontinuation of esmolol; when evaluated on an individual basis, the peak occurred 8.6 ± 2.3 minutes after the discontinuation of esmolol (Figure 2, Table 3).

**Table 3:** Time courses of heart rate and systolic and diastolic blood pressure during and after infusion of esmolol for 60 minutes on top of continuous dobutamine infusion in healthy volunteers of European ancestry (n = 16) HR: heart rate; SBP: systolic blood pressure; DBP: diastolic blood pressure; IQR: interquartile range

Time (minutes)	HR [IQR] (bpm)	SBP [IQR] (mmHg)	DBP [IQR] (mmHg)
Baseline	67.5 [63.5, 72.3]	111.5 [105.0, 114.5]	74.0 [68.8, 77.3]
-20	103.5 [95.0, 112.3]	145.0 [121.0, 153.5]	75.0 [69.5, 85]
-10	110.0 [102.8, 114.0]	142.0 [126.3, 151.0]	79.5 [71.0, 84.3]
-5	110.8 [104.8, 115.8]	138.0 [119.0, 149.3]	76.5 [71.3, 83.0]
0	113.0 [108.3, 117.0]	138.0 [120.0, 153.0]	77.0 [68.0, 83.5]
2	102.5 [91.8, 106.9]	-	-
4	84.8 [75.0, 92.8]	136.5 [121.8, 143.0]	76.0 [72.0, 884.3]
6	89.0 [73.5, 93.3]	-	-
8	84.0 [76.3, 89.3]	129.5 [122.3, 136.3]	80.0 [74.3, 84.5]
12	85.8 [74.1, 88.5]	-	-
16	81.38 [75.5, 88.3]	127.5 [119.5, 136.5]	77.5 [72.8, 83.5]
20	81.5 [73.1, 85.3]	-	-
28	81.0 [72.8, 86.8]	127.0 [120.3, 132.0]	78.5 [73.5, 81.3]
36	79.5 [73.5, 85.6]	-	-
44	79.5 [71.5, 84.3]	124.5 [118.0, 131.0]	75.0 [64.0, 82.3]
60	81.0 [73.3, 83.3]	124.5 [116.9, 128.3]	75.0 [68.8, 83.3]
62	95.3 [92.1, 101.5]	-	-
64	107.5 [101.0, 110.1]	117.5 [108.3, 126.3]	67.5 [63.0, 73.8]
66	111.5 [107.0, 114.8]	-	-
68	109.0 [105.0, 113.8]	117.5 [106.8, 127.5]	66.0 [62.8, 76.5]
72	102.5 [94.9, 109.6]	-	-
76	92.0 [85.0, 103.3]	111.0 [106.8, 124.3]	68.5 [59.8, 76.5]
80	82.5 [77.8, 94.0]	-	-
88	81.0 [75.0, 89.0]	108.5 [100.8, 116.8]	69.5 [61.8, 75.0]
96	80.0 [75.1, 88.5]	-	-
104	78.0 [73.5, 87.5]	106.5 [101.0, 112.0]	68.0 [62.0, 73.0]
120	79.5 [71.5, 85.0]	104.5 [98.5, 108.3]	67.0 [58.3, 75.0]
135	75.5 [70.5, 85.0]	106.5 [103.8, 113.3]	68.0 [59.3, 76.0]
150	74.0 [69.0, 81.0]	104.5 [101.5, 107.3]	66.3 [59.8, 72.3]
165	70.0 [67.0, 78.0]	103.0 [97.3, 109.0]	68.0 [59.8, 76.0]
180	71.0 [67.5, 80.3]	104.0 [95.30, 107.3]	65.5 [55.8, 75.3]

SBP values showed a different time course. During the titration phase of dobutamine, there was a marked increase from the baseline value of 111.5 mmHg to a maximum value of 145 mmHg. This value was observed at -20 minutes, that is, at the beginning of the dobutamine stabilization/maintenance phase. From that time point on, the SBP values decreased irrespective of the presence or absence of dobutamine and/or esmolol until 60 minutes after the discontinuation of esmolol. There was a slight downward shift of the curve between zero and eight minutes by some 8.5 mmHg in response to the initiation of esmolol administration, slightly delayed compared to the HR response. Afterward, there was a slight but continuous reduction of SBP values until the end of the esmolol infusion. When esmolol was discontinued, there was, in contrast to the HR increase, another downward shift in the curve by 7 mmHg within four minutes. The reduction continued, and baseline values were reached at 16 minutes after discontinuation of esmolol. From that time point on, the decrease to a stable plateau until the end of the observation period at around 105 mmHg was still 6 mmHg.

DBP values also showed an unexpected time course, which was however different from the one seen with SBP. There was a slight increase during dobutamine titration, but apart from a single reduced value at four minutes, values were increased during esmolol administration compared to zero minute until 26 minutes. Thereafter, there was a slow reduction, and DBP returned to baseline values by 60 minutes. A marked notch (-9 mmHg within eight minutes) occurred in response to esmolol discontinuation followed by a slight recovery (coincident with the end of the HR rebound peak) to values around 67 mmHg, which were maintained until the end of the observation period.

Safety and tolerability

During the study period, 38 AEs occurred in 12 subjects; 20 AEs occurred before the administration of esmolol. Only one AE (fatigue after esmolol administration) was considered related to the study treatment. No serious AEs were observed.

## Discussion

In this prospective study among healthy Caucasian volunteers, we evaluated the pharmacodynamic and pharmacokinetic profile of esmolol in the presence of a continuous dobutamine infusion and compared the results with those obtained in the absence of dobutamine.

Pharmacokinetics

Esmolol showed the expected pharmacokinetic behavior in the presence of dobutamine.

The geometric mean maximum concentrations of esmolol in blood reached 151 ng/ml at t_max_ of 20 minutes (median; range: 6-44 minutes). While this is close to the mean steady‑state concentration (c_ss_) of 164 ng/ml reported by Sum *et al.* at the same infusion rate [11], the c_ss_ values obtained in our study (115 ng/ml) are substantially lower. The mean volume of distribution (V_D_) reported by Sum *et al.* (2,140 ml/kg) for the same dose [11] is halfway between our results obtained with and without dobutamine (1,700 and 2,500 ml/kg, respectively).

After the end of esmolol infusion, the drug disappeared rapidly from the blood with a half-life of 2.8 minutes [Geometric mean (geo mean); 95% CI: 2.0-4.1 minutes]. This value was similar to the mean distribution half-life of 3.69 minutes at 50 μg/kg/min reported by Sum *et al.* using a model-independent kinetic analysis [11]. It is worth noting that in the study by Sum *et al.,* the distribution and elimination phases of esmolol could not be clearly resolved at infusion rates of 50 and 150 μg/kg/min [11]. Distribution and elimination half-lives could therefore be calculated for the highest dose of 400 μg/kg/min only, using the pharmacokinetic model developed by the authors, with respective values of 2.03 and 9.19 min. This is in line with our observations that distribution and elimination occur simultaneously at the dose of 50 μg/kg/min, as seen in the majority of subjects.

Geometric mean total clearance of esmolol (432 ml/kg.min) was about 20% higher than the value reported by Sum *et al.* for an infusion rate of 50 μg/kg/min (363 ± 184 ml/kg.min) [11] and more than 50% higher compared to the ones we have assessed in a similar population using both 50 and 200 µg/kg/min (Table 2). If esmolol is in fact metabolized predominantly by esterases found in the cytosol of red blood cells, as has been reported repeatedly, the higher esmolol clearance observed in our setting could be explained by higher esmolol cleavage in the erythrocytes and an increased extraction due to elevated liver passage.

Esmolol when studied *in vitro* shows a half-life of 60.4 minutes in red blood cell suspensions [18]. Plasma contribution to esmolol elimination is even lower since the *in vitro* half-life of esmolol in plasma is >180 minutes [18]. Given the* in vivo* half-life of nine minutes, the assumption that esmolol is metabolized by red blood cell esterases cannot be upheld, and it is more likely that esmolol is in fact metabolized predominantly by tissue esterases and to a far lesser extent by plasma and red cell esterases. There are several lines of evidence that support this hypothesis.

1. Marked differences in concentration between arterial and venous blood samples have been reported earlier, which indicates a substantial cleavage of esmolol during an arterio-venous passage [20,24]. It has been discussed earlier that this arterio-venous gradient may be due to the hydrolysis by tissue esterases [20].

2. A higher participation of elimination through the liver seems not plausible since the liver plays no role in esmolol elimination [25].

3. Our finding that the increase in HR and blood pressure, and, although not measured but described and obvious, also the associated increase in cardiac index leads to a reduction of passage times and increase of passage cycles per time unit. This speeded up the elimination of esmolol. Dobutamine does not necessarily increase the elimination of short-acting β-blockers, as landiolol showed no or only a minimal decrease in half-life when administered on top of dobutamine [22]. As landiolol has a substantially lower distribution volume than esmolol [8], this discrepancy can be explained easily. The *in vitro* half-life of landiolol in human blood is identical to the *in vivo* half-life [26], which indicates that landiolol is cleaved by blood esterases and does not depend on tissue esterases. In the case of esmolol, the discrepancy between the *in vitro* and the *in vivo* half-lives is however 27 to 9 minutes [19], that is, a factor of 3. This indicates that about 66% of esmolol elimination does not occur in erythrocytes but in the tissue. An increase of tissue passage cycles per time unit will accordingly accelerate the elimination of esmolol substantially.

Pharmacodynamics

Dobutamine induced a substantial increase in HR from 67.5 to 113.0 bpm. In the presence of dobutamine, esmolol induced a rapid decrease in HR. We observed a clear inverse relationship between β-blocker concentration and the decrease in dobutamine-stimulated HR. Within four minutes of infusion start, esmolol concentrations in blood increased rapidly, and HR dropped as fast. Conversely, after the discontinuation of the β-blocker infusion, HR promptly returned to its initial (dobutamine-induced) high level. After dobutamine switch-off, the HR decreased but remained elevated when compared to the pre-dobutamine levels. We suspect that the fast recovery of the HR is driven not only by the renewed beta-adrenergic stimulation by dobutamine following esmolol elimination but also by a reflex phenomenon triggered by the reduced blood pressure and a rebound effect due to the pharmacochaperoning activity of esmolol [6] as observed in an earlier study [8].

Dobutamine likewise also induced an increase in SBP and - to a lesser degree - in DBP. Within eight minutes of the initiation of esmolol administration, there was a marked SBP reduction (by some 8 mmHg) followed by a continuous decrease by another 5 mmHg during the 60-minute administration time. Of note, discontinuation of esmolol administration did not result in a recovery to pre-administration levels; on the contrary, there was a further marked decrease by some 7 mmHg within a few minutes followed by a continuous reduction towards and below the baseline level over the next 20 minutes. The effect on DBP was marginal.

While a decrease in blood pressure is common with β-blocker administration, two observations deserve mention: first of all, the time constant of the effect on blood pressure is markedly different from the one on HR. While the major part of the effect on HR (about 80%) was seen within four minutes, the effect on SBP was delayed (50% of the effect within eight minutes, and another 50% during the 60-minute administration phase). The second - and more intriguing - observation is the fact that the blood pressure did not recover after the discontinuation of esmolol administration. A rapid onset of the bradycardic effect and a delayed but sustained blood pressure-lowering effect has been described earlier for esmolol [27]. Further factors for a β-receptor-independent blood pressure-lowering effect of esmolol come from the Na, Ca, and K channel-blocking activities and the decrease in renin described for this drug [3-5,28].

In our case, one could speculate that dobutamine-induced receptor desensitization is involved in the non-recovery of SBP, but this is ruled by the complete HR recovery after discontinuation of esmolol administration.

It should be noted that the HR remained elevated after both agents were switched off. This can be explained by a persistent blood pressure reduction, a post-esmolol phenomenon described before, that induces a sympathetic reflex [27,29]. Such a reflex might also trigger coronary spasms, which have been described after esmolol administration with the intent to speed up the termination of a dobutamine stress echo [30].

In summary, esmolol inhibits the positive chronotropic effect of dobutamine in a fast and, as expected and explainable by the short half-life, reversible fashion. The dobutamine-induced elevation of SBP was likewise reduced but not in a reversible fashion. Unexpectedly, dobutamine significantly speeds up the elimination of esmolol leading to a higher clearance and consequently lower blood levels and distribution volumes when compared to the elimination pattern in the absence of dobutamine.

## Conclusions

In the presence of dobutamine, esmolol elimination is substantially faster than without dobutamine. This accelerated elimination can be explained by higher cleavage through tissue esterases induced by dobutamine-induced increased tissue passage cycles per time unit. Esmolol infusion reversibly reduces dobutamine-induced elevation of HR. In contrast, infusion of esmolol reduces the dobutamine-induced SBP without recovery after the termination of the esmolol infusion. HR remains elevated after esmolol and dobutamine most likely because of persistent blood pressure reduction.
